# Clinical efficacy of Tuina therapy combined with traditional Chinese exercises in the treatment of symptomatic lumbar disc herniation: a multicentre randomised controlled trial protocol

**DOI:** 10.3389/fneur.2025.1497933

**Published:** 2025-01-24

**Authors:** Zhihong Fan, Shenghong Jia, Xin Zhou, Chao Li, Jiahao Shao, Xiaofeng Liu, Yitao Liao, Yenan Xu, Dandan He, Shixiang Wu, Xian Zhang

**Affiliations:** ^1^Wuxi Affiliated Hospital of Nanjing University of Chinese Medicine, Wuxi, China; ^2^Department of Spine, Wuxi Affiliated Hospital of Nanjing University of Chinese Medicine, Wuxi, China; ^3^Yueyang Hospital of Integrated Traditional Chinese and Western Medicine, Shanghai University of Traditional Chinese Medicine, Shanghai, China

**Keywords:** lumbar disc herniation, Tuina therapy, traditional Chinese exercises, randomised controlled trial, study protocol

## Abstract

**Background:**

Low back pain and lower extremity sensory and functional abnormalities are common symptoms of lumbar disc herniation (LDH), which can easily cause walking dysfunction and significantly impair the quality of life of patients. Tuina and traditional Chinese exercises (TCEs) are effective in relieving pain and restoring dysfunction, and both are often used in China as a combination of passive therapy and active exercise to ease symptoms in patients with LDH. However, the majority of current clinical trials on the treatment of LDH with Tuina or TCEs are single-centre clinical studies, and the quality of these studies is generally low. Furthermore, clear evidence of clinical efficacy as to whether Tuina combined with TCEs is superior to single TCEs for improving dysfunction and pain in patients with LDH is lacking.

**Methods/design:**

The design is a multicentre, assessor-blinded clinical randomised controlled trial. A total of 166 patients with LDH (aged 18–65 years) were recruited from four centres and randomly assigned at a 1:1 ratio to two groups: the TCE group and the Tuina combined with the TCE group. Each group received three treatments over the course of 1 week for a total of 4 weeks. The primary outcome indicator was the Oswestry Disability Index, whereas the secondary outcome indicators were the Short Form of Quality of Life Scale, the Short-Form McGill Pain Questionnaire Scale, and gait analysis. Assessments were made before the treatment, at the end of the treatment, and at the third and sixth months’ follow-ups. Gait analysis was only used for comparison between the two groups before and after treatment, and did not involve follow-up. Adverse events occurring during the trial were faithfully recorded.

**Conclusion:**

The results of this study are expected to provide a more effective research protocol for symptomatic LDH and an evidence-based rationale for the efficacy and safety of Tuina combined with TCEs in the treatment of symptomatic LDH.

**Clinical trial registration:**

https://www.chictr.org.cn/showproj.html?proj=209956, identifier ChiCTR2300077361.

## Introduction

Lumbar disc herniation (LDH) is a prevalent degenerative condition of the spine, manifesting clinically as low back pain (LBP) and leg pain (1). Epidemiological studies have demonstrated that LBP is one of the 10 leading causes of disability in over 100 countries (2). LDH mostly occurs in middle-aged and young people; with the change of social lifestyle, the incidence of LDH is gradually rising, and a trend of rejuvenation is observed (3). LDH is associated with severe disability, often affecting people’s normal work and life and increasing the economic burden on individuals and society (4).

Currently, the treatments for LDH are categorised into surgical and nonsurgical. Considering effectiveness and safety, nonsurgical treatments have become the first choice for most patients, and about 90% of the patients can obtain significant improvements (5, 6). Nonsurgical treatments for LDH are varied and include medications, acupuncture, traction, physical therapy, and functional exercises; nonsteroidal anti-inflammatory drugs (NSAIDs) are often used as the first line of treatment for LDH (7). NSAIDs are effective in relieving pain but do not improve other symptoms that alleviate LDH; NSAIDS also cause gastrointestinal adverse effects (8, 9). In the pursuit of reducing treatment costs and enhancing therapeutic efficacy, nonpharmacological and complementary therapies such as acupuncture, Tuina and exercise have garnered significant attention (3).

Tuina therapy, a traditional Chinese message therapy, is recommended as a treatment modality for LDH in the guideline due to its significant clinical efficacy, low incidence of serious adverse effects, and low cost (3, 10). A preliminary study on the effect of Tuina on lumbar spine function in patients with LDH found that patients’ visual analogical scale (VAS) scores decreased, lumbar curve index and intervertebral space height increased, and lumbar sagittal Cobb angle increased after undergoing manipulation intervention for 2 weeks, showing significant clinical efficacy (11). Tuina can improve muscle tension, eliminate edema, promote blood circulation, relieve aseptic inflammation, relax muscles and relieve pain by rubbing, pinching and pressing on local tissues (12, 13). Although Tuina can relieve pain, it does not significantly improve the strength of the lower back muscles (13). Therefore, massage therapy can be used in conjunction with exercise therapy, in addition to being used alone. Some studies have demonstrated that the combination of exercise therapy and massage therapy has a positive clinical effect on the management of LBP and the restoration of function (14, 15). In China, Tuina therapy in conjunction with traditional Chinese exercises (TCEs) is often recommended to improve pain and dysfunction in patients with LDH (16).

TCEs are effective nonsurgical treatments for improving the physical and mental health of patients with chronic diseases and can provide significant relief for LBP (17). TCEs enhance spinal stability and chest and lumbar muscle strength to relieve lumbar pain, improve dysfunction, and regulate the patient’s physical and mental health (18–20). In contrast to exercise therapy, TCEs necessitate conscious inhalation and exhalation, as well as the relaxation of mental will, in addition to the appropriate physical exercise, to prevent and treat diseases (21). A systematic review revealed that breathing and meditation, emphasised by TCEs, may be one of the neurophysiological mechanisms of LBP relief in TCEs, in addition to the regulation of strength and flexibility training and pain-related brain networks in TCEs (22). Various types of TCEs include Taiji, Baduanjin, Wuqinxi, and Yijinjing (23). However, TCEs are complex and variable in their movements, and lack a collection of movements that are easily learned and suitable for exercise in individuals with LDH. To identify suitable exercises for LDH patients, an expert questionnaire survey was conducted through the Delphi method. This process involved the screening of four movements from Taiji, Baduanjin, and Wuqiuquan.

At present, clinical trials investigating the nonsurgical treatment of LDH are typically characterised by a limited number of participants and a low level of methodological rigour (24). No clear current evidence of clinical efficacy suggests that treatment with Tuina in combination with TCEs is more effective than TCEs alone in improving pain and dysfunction in patients with LDH. Consequently, the present study proposes to conduct a broader validation study (multicentre, randomised, controlled and assessment-blinded clinical trial) by combining Tuina with TCEs. This approach will be achieved using clinical assessment and gait analysis to validate that combined therapy plays a superior role in improving dysfunction, pain and gait in patients with LDH.

## Methods

### Study design

**Figure 1:**
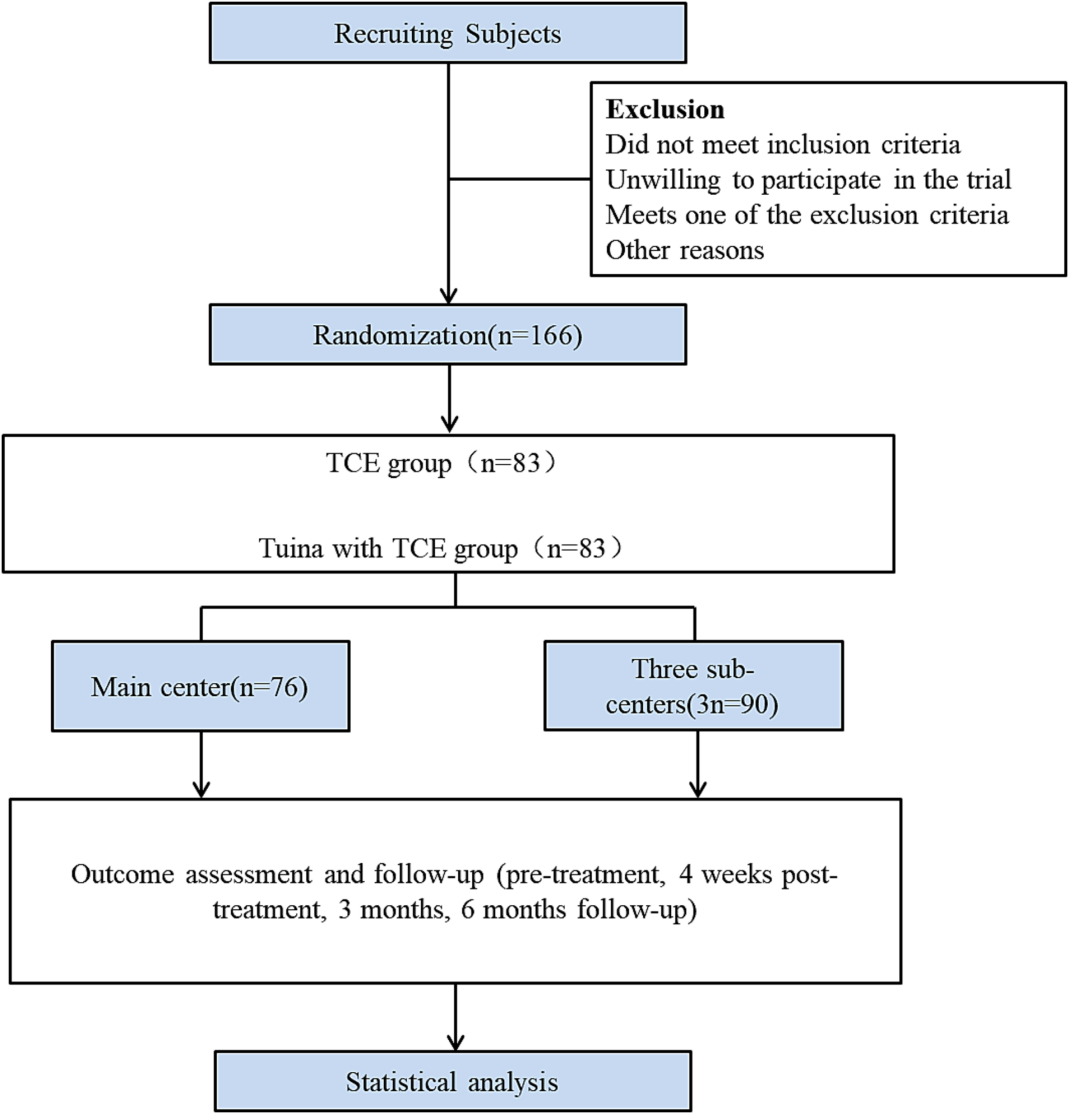
Flow chart of the pilot study.

This study is a multicentre randomised controlled clinical trial study conducted in four clinical centres (Figure 1). The Wuxi Hospital of Traditional Chinese Medicine is the main centre. The Yueyang Hospital of Integrative Medicine affiliated with the Shanghai University of Traditional Chinese Medicine, Wuxi Rehabilitation Hospital and the Wuxi Xinwu District Hospital of Traditional Chinese Medicine are the three subcentres. Patients were recruited through advertisements at each centre. Patients who met the inclusion criteria were randomly assigned to either the TCE group or the Tuina combined with TCE group at a 1:1 ratio. The trial consisted of a four-week intervention period and subsequent three- and six-month follow-up periods. Data were collected before treatment, after 4 weeks of treatment, and at 3 months and 6 months of follow-up. The outcome assessment indicators included the Oswestry Disability Index (ODI), Short Form of Quality of Life (SF-36) Scale, Short-Form McGill Pain Questionnaire (SF-MPQ) Scale and gait analysis, which was used only for efficacy evaluations before and after 4 weeks of treatment.

### Ethics approval

The study was approved by the Ethics Committee of the Wuxi Hospital of Traditional Chinese Medicine (STHZG2023021301), and it was registered in the China Registered Clinical Trial Registration Centre (ChiCTR2300077361). The study strictly followed the criteria outlined in the Declaration of Helsinki, the Consolidated Standards of Reporting Trials (COMSORT) and the Standardised Program Item: Recommendations for Interventional Trials (SPIRIT) guidelines (25).

### Recruitment

Subjects were recruited at each subcentre through a variety of channels, including printed advertisements, flyer distribution, telephone inquiries, and public announcements on the hospital’s WeChat public website. Each subject participating in the trial must meet the inclusion criteria. Subjects who meet any of the exclusion criteria subject will be excluded. The principal investigator will not request that the subject sign an informed consent form until the subject has had sufficient opportunity to understand the nature of the trial and to agree to participate in it.

### Inclusion and exclusion of subjects

#### Inclusion criteria

1. Age ≥18 and ≤65 years old, regardless of sex.
2. Meeting the diagnostic criteria for LDH (26).
3. Duration of the disease ≥3 months.
4. VAS scores >3 and ≤7.
5. Voluntarily participation and signed informed consent form.

#### Exclusion criteria

1. Patients with a history of previous severe spinal trauma.
2. Patients with spinal bone tumours, tuberculosis and osteoporosis, as seen on imaging.
3. Patients with severe neurological deficits, such as cauda equina injury.
4. Patients with a combination of cardiovascular, cerebrovascular, hematopoietic, gastrointestinal and other serious illnesses or psychiatric disorders.
5. Patients with other autoimmune diseases, metabolic disorders, and acute and chronic infections.
6. Patients who have participated in other clinical trials.

Those with one of the above conditions cannot be included in this trial.

### Randomisation, allocation concealment and blinding

The random sequence was generated by a statistician independent of the study through a central randomisation system. The random numbers and group assignments were provided to the independent researcher through the central randomisation system. This researcher was not involved in any other aspect of the trial. The physician only obtained the grouping information from this independent researcher by telephone before the treatment. The central randomisation system had strict limitations on the permissions granted, and only the supervisory staff had access to these documents. This procedure ensured adequate concealment of the randomisation. The study was not blinded to the patients and therapists, but it was blinded to the evaluators. This evaluator was independent of this study. The TCEs therapists were separated from the Tuina therapists, and neither group was aware of the grouping of the patients. Patients were not allowed to interact with the therapist beyond general counseling about their health status at each session.

### Sample size calculation

The trial was designed as a superiority trial with ODI as the primary observation index. The previous study demonstrated that the mean and standard deviation of ODI after 4 weeks of intervention in the TCEs group were 19.73 and 1.94, respectively, whereas the mean and standard deviation of ODI after 4 weeks of intervention in the Tuina combined with TCE group were 14.53 and 2.60, respectively. The sample size was calculated using the following formula:


$$n=\frac{2{\left(Z_{\alpha /2}+Z_{\beta }\right)}^{2}\times \sigma^{2}}{\left(\mu_{t}-\mu_{c}-\Delta \right)}$$

$$\mu_{t}=14.53,\mu_{c}=19.73,\alpha =0.05,\beta =0.2,\sigma =2.60,\Delta =-4$$

The sample size for each group was 74. Because this trial was a superiority trial, the sample size of each group was the same, and the clinical trial was set up in two groups (Tuina with TCE group and TCE group), combined with the relevant regulations. To evaluate the efficacy and safety of this study fully, the total number of cases was planned to be 166 cases, considering the potential for adverse effects. The Wuxi Hospital of Traditional Chinese Medicine was responsible for this project and was the main centre where the case study was undertaken. In other subcentres, the number of cases can be adjusted according to the situation, but no fewer than 30 cases should be in each group in each centre. If enrolment cannot be completed in any centre due to insufficient suitable subjects, the cases were distributed among the centres.

### Intervention

This study is a multicentre clinical trial. To ensure the consistency and standardisation of treatment modalities across all clinical centres, therapists from the main centre conducted training sessions for therapists from the subcentres. The objective of these sessions was to standardise the Tuina treatment protocols and the TCE movement, and to treat patients in accordance with the interventions outlined in the study protocol. Prior to intervening with patients, therapists at subcentres must undergo training and evaluation at the main centre. They are not qualified to intervene until they have met the training requirements. Furthermore, to mitigate bias in the treatment of patients by different therapists, only one Tuina therapist and one TCE therapist were appointed to conduct the intervention at each clinical centre. TCE therapists must have at least 10 years of experience teaching TCEs.

To ensure that each subject can master the movement essentials of TCEs, a TCE treatment manual was issued to the subjects before the intervention. A comprehensive introduction to the movement essentials and precautions of TCEs through offline lectures and online video teaching mode followed. The therapist maintained a treatment log for each subject and recorded the subject’s progress and any issue that arise. If a subject has not completed the prescribed treatment within the allotted time, the therapist will contact the subject by phone to ensure that the subject has met the standard treatment volume.

### TCE group

**Figure 2:**
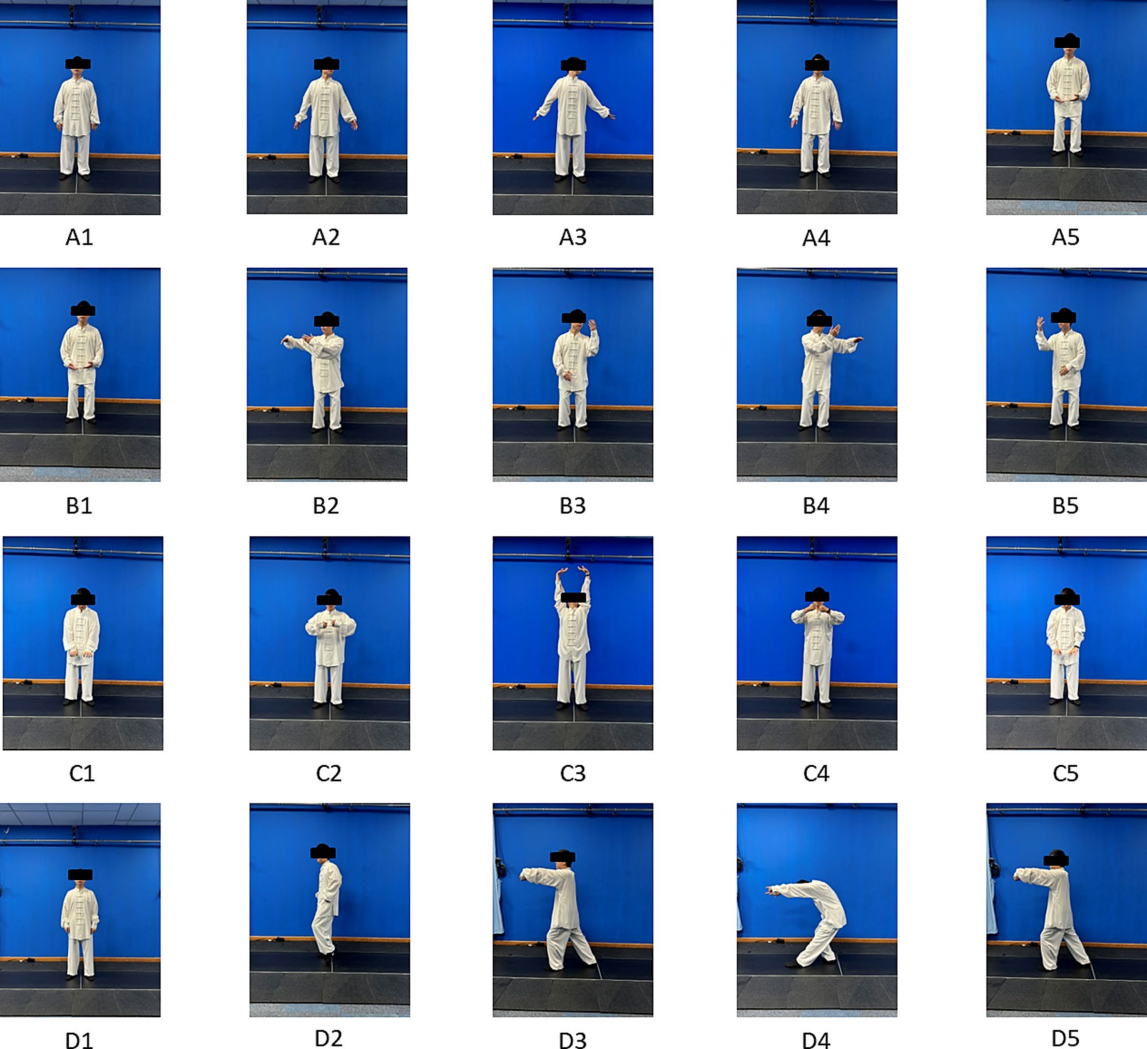
Schematic diagram of the movements of the four TCEs. **(A)** Fourth movement of Baduanjin, **(B)** Yunshou **(C)** Huju, **(D)** Luben. **(D2–D5)** Demonstrates the movements from the side. Each movement alternates between the left and right sides or the up and down directions, and the entire sequence is continuous. Each movement is repeated three times, and four movements are performed as a set for a total of two training sets.

The TCE group comprised four movements, selected from Baduanjin, Taiji and Wuqinxi, with minor modifications to accommodate the patients’ varying learning and exercise abilities. The program primarily encompassed the fourth movement of Baduanjin, the Yunshou of Taiji, Huju and Luben from Wuqinxi. All movements were supervised by qualified TCE instructors. Firstly, patients performed warm-up exercises for about 5 min to avoid muscle strain during exercise. Then, the TCEs were performed in the order of A–D (Figure 2), and each movement was repeated 3 times, with 4 movements as 1 set, for a total of 2 sets of exercise, which should last 15 min or more. Finally, a 5-min stretching exercise was performed. Treatments were performed 3 times a week for 4 weeks.

### Tuina with TCE group

**Figure 3:**
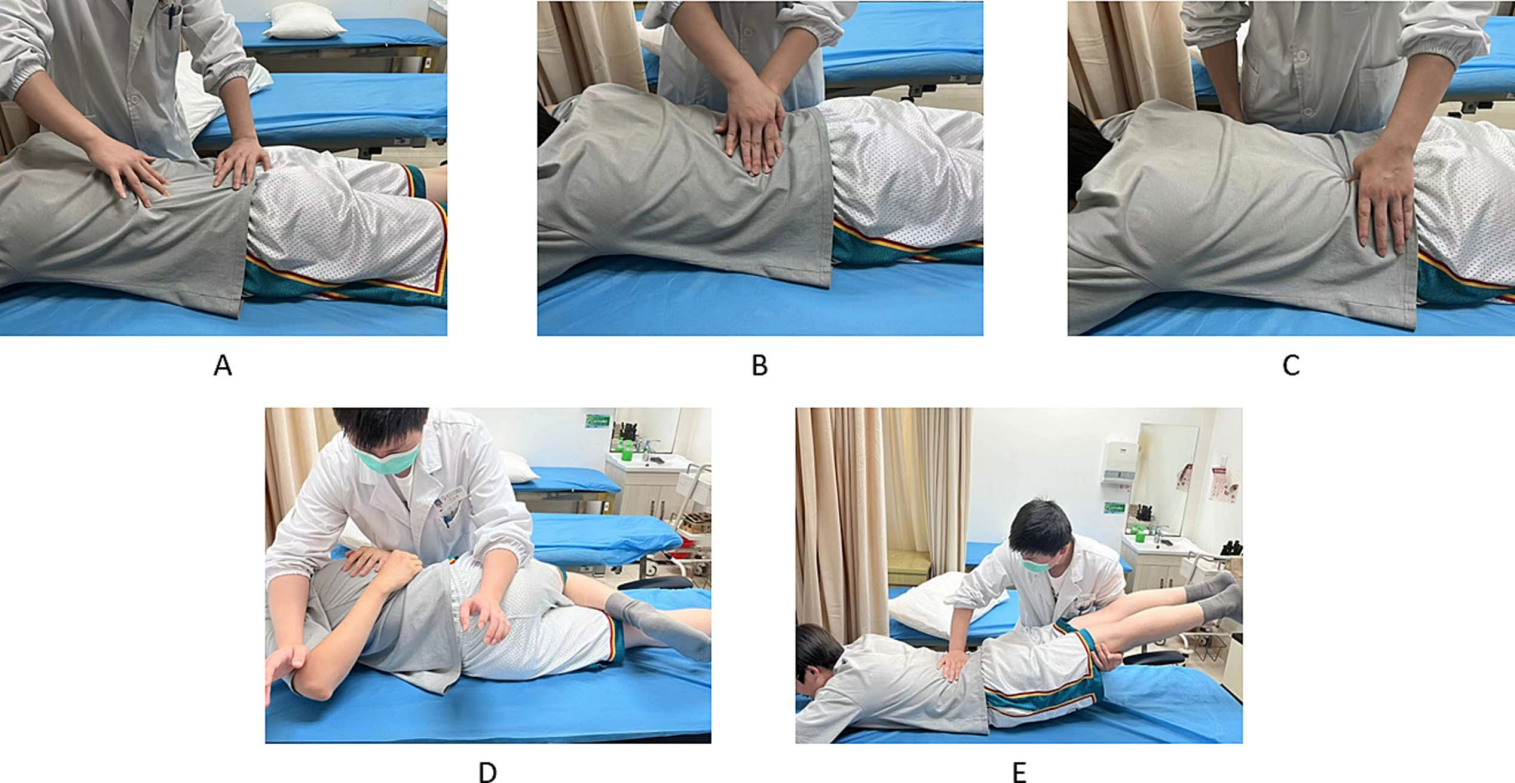
Tuina operation method. **(A–C)** Localised muscle release. **(D,E)** Lumbar joint adjustments. **(A)** Press and wipe. **(B)** Pressure and kneading. **(C)** Pressing on pain points or acupoints. **(D)** Lumbar blique-pulling manipulation. **(E)** Posterior lumbar extension.

The Tuina procedure was divided into localised muscle release and lumbar joint adjustment, and the whole process lasted for 20–30 min (Figure 3). The procedure started with gentle rubbing and kneading on both sides of the patient’s spine and buttocks using the fingertip surface for 10–15 min, and then pointing and pressing the local pain points or acupoints (e.g., BL23, BL24, BL25, BL40, and GB30) for 5 min. Following the completion of local muscle release, joint adjustments were performed utilising a lumbar blique-pulling manipulation and posterior lumbar extension. The lumbar blique-pulling manipulation was comparable with chiropractic manipulation (27), where an audible “click” indicated the successful completion of the procedure. Finally, lumbar back extension was performed. The patient was initially placed in the prone position, and the therapist pressed one hand on the patient’s spinal pain and slightly lifted the patient’s lower limbs with the other hand. The therapist then waited for the patient’s lumbar muscles to relax. Then, the therapist quickly lifted the patient’s lower limbs up to 30°–40° and then placed them down immediately. This maneuver can be done 3–5 times for about 5 min. Following Tuina, the patient should be permitted to rest in bed for approximately 10–15 min. The training movements and methods employed by the TCEs were identical to those utilised in the control group. Treatments were performed 3 times a week for 4 weeks.

### Outcomes

**Figure 4:**
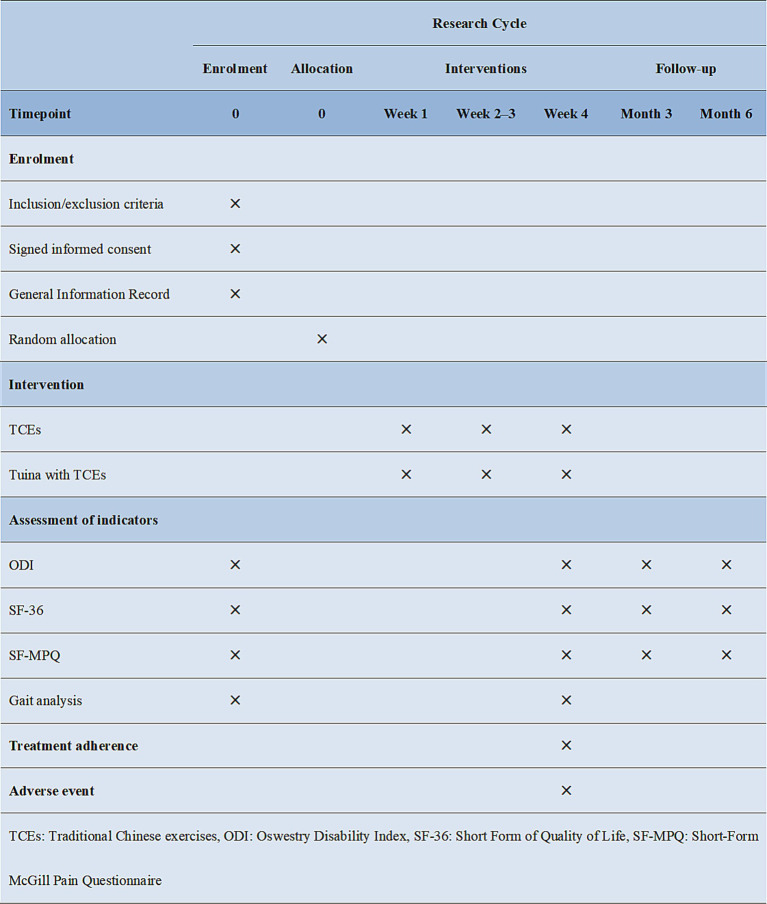
Enrolment, pre- and post-intervention, and follow-up data collection schedules.

The efficacy evaluation in this study concentrated on the primary outcome indicator, namely the recovery of dysfunction in patients before, after and during the follow-up period. In addition, several secondary indicators were evaluated, including quality of life, pain and gait analysis. The data collection at each time point was conducted by independent researchers who were not privy to the specific subgroups and were not involved in other aspects of the study. The specific time points for data collection are presented in Figure 4.

### Primary outcome

The ODI scale, which consists of 10 items in 3 dimensions, namely pain, individual functioning and overall personal functioning, was used as a comparison of efficacy before and after treatment and between groups (28). The maximum score was 50; the higher the score, the higher the degree of dysfunction of the patients.

### Secondary outcome

The SF-36 Scale evaluates health in eight dimensions, including physical functioning, general mental health and social functioning, and higher scores indicate better health (29). Each dimension was rated with a maximum of 100 points and a minimum of 0 points.

The SF-MPQ Scale includes three parts: pain rating index (PRI), (VAS) and present pain intensity (PPI) (30). The PRI assesses the patient’s emotional and sensual experiences of pain. The VAS provides a straightforward visual representation of the patient’s pain level. The PPI determines the intensity of the patient’s current pain on a six-point scale (0–5).

Gait analysis is the process of collecting patient information using a wearable 3D gait analyser. Gait analysis encompasses a range of patient data, including step length, cadence, stride length and double support phase. This information is utilised to assess the patient’s motor function and musculoskeletal rehabilitation status (31).

After all the relevant items were confirmed by the patients themselves, the total score reflecting the degree of low back and leg pain in each period was calculated cumulatively by a specialised statistician, and the results were used for pre- and post-treatment comparisons as well as comparisons between groups.

### Adverse events

The treatment responses of all patients with lumbar intervertebral disc herniation who received Tuina in conjunction with TCEs were monitored and documented throughout the course of the study. Adverse events during the treatment period were recorded and analysed to ascertain whether it was related to the treatment. Adverse events included those that occurred during the clinical trial and resulted in hospitalisation, prolonged hospitalisation, disability, impairment of the ability to work, life-threatening injury or death. In case of any serious adverse event, whether related to the intervention, the trial should be terminated immediately and appropriate rescue treatment initiated without delay. Such incidents must also be reported within 24 h to the responsible unit, the ethics committee, the sponsoring organisation and the relevant personnel of the drug regulatory authority. The relevant information must be recorded in the case file.

### Quality control

Prior to the implementation of the trial, ensuring that all study personnel received a uniform, centralised training was necessary to achieve an accurate understanding and mastery of the various criteria for cases (diagnosis, inclusion, exclusion, exclusion, shedding and termination criteria), the technical protocols of the treatment, the observational indicators and the scoring criteria. The case report form (CRF) was completed in its entirety and in accordance with the prescribed specifications, with no arbitrary alterations. Furthermore, observations of the same subject were recorded by the same individual whenever feasible.

### Data collection and management

A paper-based case record form was employed for data collection. The healthcare professionals proactively cooperated with the patients to complete the assessment of each outcome indicator and tolerantly answered the patients’ questions related to each assessment scale. Feedback was provided to patients promptly following each outcome assessment. The sub-centers used a uniform case record form for data collection. The case record forms were then reviewed by the subject leader and research assistant before submission to the data manager for data entry and management. The data were entered and managed by the data managers using an Excel database. To ensure the accuracy of the data, double-entry assessment and proofreading was conducted independently by the two data managers. In the event of uncertainty regarding the data entry on the case record form, the data administrator will complete the question-and-answer form and forward a query to the individual responsible for the subject matter through the research assistant. The individual responsible for the subject matter should provide a response as soon as possible. The data administrator will then modify, confirm and enter the data in accordance with the response provided by the individual in charge of the subject matter. Once the database has been confirmed to be error-free, it will be locked by the subject person in charge, the data manager and the statistical analyst. The data collected from subjects will not be made public without their consent.

### Statistical analysis

The data will be subjected to analysis by our team of professional statisticians, who will employ IBM SPSS Statistics V.27.0 for this purpose. To assess the normality of the data, the Kolmogorov–Smirnov test was used. The quantitative results were expressed in terms of the mean and standard deviation. The purpose of the primary and secondary outcome indicators was to assess the difference in efficacy between the two groups. Hypothesis testing was employed to this end. The chi-square test was used to ascertain the dissimilarities in baseline data between the two groups. The analysis of variance for repeated measures was utilised to examine the alterations in efficacy between the two groups before, during and after the intervention, as well as after the follow-up period. For confounding factors that were challenging to regulate or were not regulated prior to treatment, such as an imbalance between the groups prior to treatment, were treated as covariates, and logistic regression was employed to ascertain the variability of efficacy between groups and to eliminate the influence of these factors on efficacy. The data collection and data summarisation were meticulously examined to minimise data anomalies. In the event of missing data, the data were assumed to be missing at random, and multiple interpolation would be employed for analysis. Subjects who were excluded from the trial will be excluded from the data set. The level of statistical significance was set at 5% (*p* < 0.05), and 95% confidence intervals were employed as effect estimates.

## Discussion

Tuina and TCEs are an important part of Chinese traditional medicine with a history of thousands of years, playing an important role in protecting the health of the Chinese people and accumulating a great deal of clinical experience. Although multicentre clinical studies have been published on the use of Tuina combined with TCEs for the treatment of LDH (16), they have not performed long-term observations of efficacy, and the TCE manoeuvre lacks specificity. Standardised clinical randomised controlled trials to validate whether Tuina combined with TCEs is necessarily superior to single TCEs for relieving dysfunction and pain in patients with LDH are also lacking. Consequently, a multicentre, large-sample, assessor-blinded clinical randomised controlled trial was designed. The short- and long-term improvement of dysfunction and pain in LDH patients with Tuina combined with TCEs was assessed by evaluating the health status of the subjects through multiple outcome indicators at preintervention, after 4 weeks of intervention, and at the third and at sixth months’ follow-up to validate that the combination therapy is more advantageous than the treatment with single TCEs.

This study was conducted at multiple clinical centres, offering several advantages. Firstly, this study used four clinical centres, divided into one main centre and three subcentres. This structure facilitated more opportunities for subject recruitment and ensured an adequate sample size. Secondly, the results can be independently verified and repeated in each subcentre to confirm the consistency and reliability of the main results, thus reducing bias and increasing credibility. Thirdly, the observations focused on the recovery of dysfunction in patients with LDH, which was assessed using gait analysis to assist in evaluating the recovery of walking function in addition to the ODI scale. Additionally, a follow-up period of up to 6 months was conducted following the four-week intervention to facilitate observation of the short- and long-term efficacy of Tuina combined with TCEs for LDH.

In addition to these advantages, this study had some shortcomings. Firstly, due to the constraints of the intervention, blinding the treating physicians and the patients was not feasible. Consequently, any potential bias could only be mitigated by blinding the assessors. Secondly, the majority of the outcome indicators were subjective evaluators (e.g., ODI, SF-36, and SF-MPQ), which were vulnerable to patients’ subjective awareness, placebo effects and anticipated outcomes. Despite these limitations, these shortcomings will be addressed in future studies. This study will provide scientific evidence-based medical evidence for the efficacy and safety of Tuina combined with TCEs to improve dysfunction and pain in patients with LDH, and a more cost-effective, safe and effective treatment for LDH.
